# Adaptive divergence of the moor frog (*Rana arvalis*) along an acidification gradient

**DOI:** 10.1186/1471-2148-11-366

**Published:** 2011-12-19

**Authors:** Sandra Hangartner, Anssi Laurila, Katja Räsänen

**Affiliations:** 1 EAWAG, Department of Aquatic Ecology, and ETH-Zurich, Institute of Integrative Biology, Ueberlandstrasse 133, CH-8600 Duebendorf, Switzerland; 2 Population and Conservation Biology/Department of Ecology and Genetics, Evolutionary Biology Center, Uppsala University, Norbyvägen 18D, SE-752 36 Uppsala, Sweden

## Abstract

**Background:**

Environmental stress can result in strong ecological and evolutionary effects on natural populations, but to what extent it drives adaptive divergence of natural populations is little explored. We used common garden experiments to study adaptive divergence in embryonic and larval fitness traits (embryonic survival, larval growth, and age and size at metamorphosis) in eight moor frog, *Rana arvalis*, populations inhabiting an acidification gradient (breeding pond pH 4.0 to 7.5) in southwestern Sweden. Embryos were raised until hatching at three (pH 4.0, 4.3 and 7.5) and larvae until metamorphosis at two (pH 4.3 and 7.5) pH treatments. To get insight into the putative selective agents along this environmental gradient, we measured relevant abiotic and biotic environmental variables from each breeding pond, and used linear models to test for phenotype-environment correlations.

**Results:**

We found that acid origin populations had higher embryonic and larval acid tolerance (survival and larval period were less negatively affected by low pH), higher larval growth but slower larval development rates, and metamorphosed at a larger size. The phenotype-environment correlations revealed that divergence in embryonic acid tolerance and metamorphic size correlated most strongly with breeding pond pH, whereas divergence in larval period and larval growth correlated most strongly with latitude and predator density, respectively.

**Conclusion:**

Our results suggest that *R. arvalis *has diverged in response to pH mediated selection along this acidification gradient. However, as latitude and pH were closely spatially correlated in this study, further studies are needed to disentangle the specific agents of natural selection along acidification gradients. Our study highlights the need to consider the multiple interacting selective forces that drive adaptive divergence of natural populations along environmental stress gradients.

## Background

The many ongoing environmental changes, such as global climate change, use of agrochemicals and invasion of new species, result in stressful conditions, which challenge the persistence of natural populations and reduce biological diversity [e.g., [1]]. However, environmental stress, an environment that lies outside the range of preferred conditions of an individual and challenges an organism's ability to maintain function [2], can also be a powerful evolutionary force [e.g., [3,4]]. For instance, stressful environments can cause selection either directly on organismal stress tolerance, or indirectly - and interactively - through correlated ecological changes [e.g., [5]] on organismal growth and development rates [6]. In line with geographic variation in local environmental conditions causing divergent natural selection, and facilitating local adaptation [7-9], empirical work has found evidence for local adaptation to environmental stress, such as salinity, acidity, heavy metals or temperature [10-13]. However, what are the drivers of divergence along environmental stress gradients in natural populations remains little studied.

Chemical environmental changes are an obvious source of environmental stress, and increasingly impact natural populations [e.g., [4,14,15]]. One of these is natural and human induced acidification, which has strong lethal and sub-lethal (e.g. reduced growth) effects on individuals in a broad range of taxa [16-18], and may cause strong natural selection [e.g., [13]]. Accordingly, there is some evidence for evolution of increased acid stress tolerance and adaptive divergence from zooplankton [e.g., [19]], fish [e.g., [20]] and amphibians [e.g., [13,21]].

We here studied phenotypic divergence of the moor frog, *Rana arvalis*, along an acidification gradient in Sweden. In amphibians, environmental acidity increases embryonic mortality and slows down larval growth and development, resulting in delayed metamorphosis at smaller size [reviewed in [17]]. As both embryonic survival and metamorphic traits are important fitness components in amphibians [e.g., [22,23]], acidity should cause strong divergent selection on these traits. In accordance, in *R. arvalis*, evidence for adaptive divergence has been found in embryonic acid tolerance [13,24-26], and a recent study on two populations suggested that acidity may select for faster growth, slower development and larger metamorphic size [21]. However, to what extent larval trait divergence reflects a general pattern in response to pH mediated selection, and to what extent adaptive divergence occurs in larval stress tolerance (as reflected in genotype × environment (G × E) interactions [27]) is not known. We therefore conducted common garden laboratory experiments to study the extent of divergence in embryonic and larval acid stress tolerance, and larval life-history traits among eight *R. arvalis *populations along an acidity gradient (breeding pond pHs from 4.0 to 7.3).

Many putative selective agents can co-vary in nature, either as a result of correlated changes in response to a main driver, or due to interactions with existing locally varying environmental differences. These then create local variation in combinations of different selective factors. For instance, in addition to declines in pH, environmental acidification leads to several abiotic and biotic changes - such as leaching of metals, reduced humic compounds, increased UV-B radiation, changed population densities and species composition [28]. To test for indirect evidence of selection using phenotype-environment correlations, we measured pH and a range of abiotic and biotic environmental variables at each site. In addition to inferences about putative agents of selection along environmental gradients, these measurements allow hypotheses about variation in the strength of divergent selection among natural populations - where estimating the strength of divergent selection is otherwise difficult [e.g., [29]]. In particular, the strength of divergent selection is expected to correlate with the magnitude of environmental differences [e.g., [9,29,30]].

We predicted that 1) if populations have diverged in acid stress tolerance, acid origin populations should show higher acid stress tolerance (reflected in G × E interactions) than populations from more neutral sites, 2) if populations have diverged in larval life histories, we should see contrasting patterns between acid and neutral origin populations in growth and development rates and metamorphic size, and 3) if environmental acidity (or something closely correlated with it) reflects the agent and strength of selection, phenotypic trait values should correlate with breeding pond pH. Any deviations from these patterns would suggest that acidity *per se *is not the most important selective force along this acidity gradient, or that there are constraints (e.g., trade-offs or gene flow) to adaptation.

## Methods

### Study species and populations

*R. arvalis *is a ranid frog that occurs in the western Palaearctic in a wide range of habitats and acidity levels [31]. Breeding occurs in early spring, at higher latitudes soon after spring snow melt, hence coinciding with peak acidity in acidified areas.

**Figure 1:**
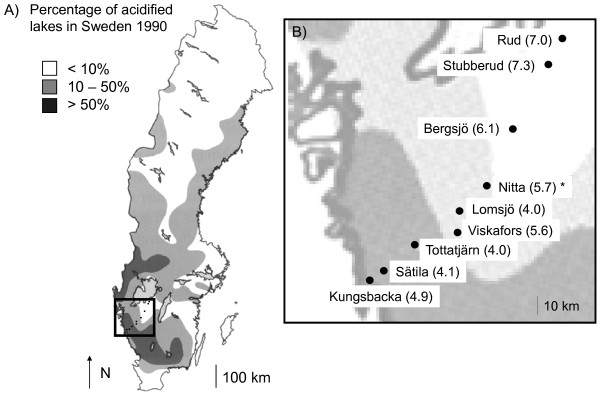
**Map of the location of the study and the study ponds**. Map of Sweden showing A) the location of the study region (square) and study ponds (black dots) in relation to geographic variation in anthropogenic acidification in 1990 and B) the study region with nine populations and their pond pHs (in brackets). The pond Nitta (*) was only used for environmental variation. (Source: Swedish Environmental Protection Agency: http://www.naturvardsverket.se/en/In-English/Start/State-of-the-environment/Acidification/.

Eight populations occurring along a ca. 160 km transect in southwestern Sweden were used in this study (Figure 1, Table 1). All study sites are permanent ponds or small lakes in forested areas (Figure 1, Table 1). Breeding pond pH among these sites ranges from pH 4.0 to 7.3. The exact acidification history of these ponds is not currently known, but is likely influenced by a mix of anthropogenic [acid rain, [28,32]] and natural acidification (Table 1), which may have been counteracted in some locations with artificial liming [33]. The two most neutral sites (Figure 1, Table 1) are buffered naturally due to limestone bedrock [34].

**Table 1:** Descriptive information on nine study ponds along a pH gradient.

Population	A)	B)	C)	D)	E)	F)	G)	H)	I)	J)	K)
Tottajärn	100	7	57°60N, 12°60E	141	40	4.0 ± 0.2	Natural & human	15.5	6.0	0.2	462683
Lomsjön	50	11	57°76N, 12°88E	268	20	4.0 ± 0.2	Natural & human	14.6	14.2	0.7	284400
Sätila	45	6	57°51N, 12°34E	94	30	4.1 ± 0.2	Natural & human	15.1	9.7	0.1	263647
Kungsbacka	80	8	57°50N, 12°06E	46	60	4.9 ± 0.2	Natural & human	17.0	10.2	0.2	65120
Nitta*	> 500		57°87N, 13°21E	240	70	5.7 ± 0.3	Unknown	14.2	2.0	5.7	286853
Viskafors	270	6	57°65N, 12°87E	146	60	5.6 ± 0.3	Indirect liming	14.4	6.1	1.4	754776
Bergsjön	> 500	10	58°20N, 13°48E	310	10	6.1 ± 0.3	Unknown	14.8	4.7	8.6	7221305
Stubberud	250	9	58°46N, 13°76E	281	40	7.3 ± 0.2	Limestone area	14.8	7.0	2.9	34128
Rud	300	10	58°59N, 13°76E	88	30	7.0 ± 0.2	Limestone area	15.4	6.5	2.2	267701

The A) approximate numbers of breeding *R. arvalis *females, B) number of full-sib families used per population, C) coordinates (N, E), D) altitude (m), E) forest canopy closure (%), F) mean ± SD pond pH, G) likely acidification history, H) average temperature (°C) during March - July 2009, I) predator density (per five sweeps), J) amphibian larval density (per five sweeps), and K) pond size (m3). Number of breeding females is based on clutch counts in 2007-2009. *Study site was only used for environmental information. Pond pH is based on averages of three sites within each pond in April 2008 and April, May and June in 2009.

### Quantification of the selective environment

To characterize environmental differences among breeding ponds, pH and several abiotic and biotic variables known to change as a result of acidification, or to be ecologically important for amphibians, were measured (Table 1). The abiotic environment was characterized as pH, latitude and altitude, water temperature, pond size and canopy cover. The biotic environment was characterized as predator abundance and tadpole density. These variables were chosen because seasonal time constraints and temperature can have strong effects on larval life history traits in amphibians [35], canopy cover and pond size may affect amphibian growth and development [36,37], and community composition typically shifts towards more insect dominated predator communities and reduced amphibian densities as a result of acidification [28]. To increase sample size for environmental inferences, habitat variables were also measured in one additional population, which was, however, not included in the experimental work (Nitta, Table 1).

pH was measured annually since 2007, whereas the other environmental variables were quantified in 2009. pH was measured in April 2007 to 2009, and May and June 2009 with an Orion 9109WL electrode (Thermo Scientific, Inc.) attached to a portable field pH-meter (Orion 260, Thermo Scientific, Inc.). At each pond, pH was measured at three different sites (three measurements per site) during one to three visits/year. pH for each pond was calculated based on pH averages of April 2007-2009 (hereafter "embryonic period") and April 2007 and 2008 (no May and June data available for these years), May and June 2009 (hereafter "larval period").

Latitude and altitude were gained from Google Earth http://earth.google.com/ and used as indicators of large-scale climatic variation. Local temperature was measured with iButton data loggers (DS1921G-F50, Maxim Integrated Products, Inc.) in 2009 from April 14 (early breeding season) until June 17 (when tadpoles were approaching metamorphosis). The loggers were set at measuring interval of three hours and placed just below water surface in sealed plastic bottles (one per two or three different sites within a pond), near *R. arvalis *breeding sites. Due to technical fault, data from only one logger could be retrieved from two of the ponds (Rud and Sätila, Table 1). For each pond, the mean temperature was calculated over the whole recording period and across sites.

To get an estimate of pond size, pond area was estimated by multiplying pond length × pond width (measurements gained from Google Earth). Shoreline depth at each pond was measured in May and June 2009 (to the nearest 0.01 m) at three randomly chosen, approximately equidistant, sites (using a measuring band with a weight attached to assure a straight line). The average of May and June measurements/pond was used as pond depth. Pond size (m^3^) was then calculated by multiplying pond area (m^2^) with pond depth (m) and log-transformed for the statistical analyses. The percentage of forest canopy closure was estimated as the proportion of non-visible sky within an angle of 40°- 45° by the same person (S.H.) in 10% categories [38].

Predator and tadpole densities were estimated at each pond in mid May and mid June 2009 with dip net (32 cm diameter, 0.08 cm mesh size) sweeps. Within each pond, five sweeps at a depth of 0.5-1.5 m over a 2-3 m distance along the shoreline were made at three equidistantly distributed sites at each sampling occasion (May and June). All tadpoles (*Bufo bufo, R. arvalis *and *R. temporaria *are the only anurans occurring at these ponds) and all invertebrate (aeshnid, libellulid and zygoptera larvae, notonectid bugs and dytiscid beetles) and vertebrate taxa (fishes and adult newts, *Triturus vulgaris *and *T. cristatus*) considered as potential predators were placed in a white square plastic box and photographed for later counts of predator and tadpole numbers. All animals were subsequently released. The total number of predators and tadpoles over the five sweeps/site was counted from the photographs. The average numbers over the three sampling sites per pond in May and June were taken as predator and competitor abundance in each pond.

### Experimental design and rearing of embryos and tadpoles

Five to 11 pairs of males and females in breeding condition were collected from each of the eight populations (168 individuals in total) in spring 2008 (Table 1) and transported to the laboratory at Uppsala University (59°50'N, 17°50'E). The adults were kept in a climate chamber at +2 to 4°C until artificial crosses were made a few days later. Artificial mating prevented any bias due to differences in exposure in the early environment and assured that the offspring in each family were full sibs. The crosses were performed at +16°C according to [13,39] with some modifications. Sperm production was stimulated by injecting males with ca. 2 μg/25 g body mass of the fish hormone LHRH [H-7525, Bache Bioscience Inc., [40]]. Females were not treated with hormones, as they had already ovulated. Sperm from males was collected by rinsing their cloacae with sterile physiological salt solution into 0.9 l plastic vials containing 10% Amphibian Ringer solution [41]. Sperm motility of each male was checked under a microscope. Eggs from the females were subsequently stripped into the sperm solution [39] and treated using standard procedures [13]. The fertilized eggs were divided to the treatments two hours after fertilization but before the first cell cleavage.

The embryonic experiment consisted of five to 11 families/population (Table 1) with three pH treatments (embryos: pH 4.0, 4.3 and 7.5) and three replicates/family/treatment. The larval experiment consisted of four to 11 families/population with two pH treatments (larvae: pH 4.3 and 7.5) and nine replicates/family/treatment. These treatments were chosen as they reflect pH levels at our most extreme sites in the wild and are known to reduce embryonic survival and/or cause sub-lethal effects (reduction in growth and developmental rates) in the larvae [21,25]. During both experiments, the experimental vials were randomly distributed over three blocks (embryos: one replicate per family and treatment per block, larvae: three replicates per family and treatment per block) according to a known temperature gradient within the room. This design resulted in a total of 631 experimental units for the embryonic and a total of 1079 experimental units for the larval experiment.

Embryos and tadpoles were reared in reconstituted soft water [RSW, [42]], as described in Räsänen et al. [21]. Untreated RSW was used for the neutral (pH 7.5) treatment. RSW in the acid (pH 4.0 and 4.3) treatment was adjusted with 1 M H_2_SO_4 _over two days before use. To maintain appropriate pH and water quality, water was changed every three days in the embryonic and every two days in the larval part of the experiment. Prior to each water change, pH was measured in randomly selected experimental vials (pH 4.0 and pH 4.3 treatment: three vials; pH 7.5 treatment: one vial) with a Ross Sure-flow electrode (model 8172BN, Thermo scientific, Inc.) and a Thermo Orion 3 Star pH-meter (model 1112000, Thermo scientific, Inc.). pH drift between the water changes was low during the embryonic experiment (mean pH ± S.D. in treatments: pH 4.0 = 4.03 ± 0.05, pH 4.3 = 4.29 ± 0.08, pH 7.5 = 7.63 ± 0.08). It was also relatively low during the first half of the larval experiment (pH 4.3 = 4.49 ± 0.19, pH 7.5 = 7.43 ± 0.13). During the second half of the larval experiment, pH deviated more strongly due to increasing tadpole biomass and food (pH 4.3 treatment = 4.96 ± 0.17, pH 7.5 treatment = 7.14 ± 0.09). However, the low pH treatment always remained clearly acid (below pH = 5.5) and the two treatments never overlapped.

The experiments were conducted in a walk in climate room (+16°C) with a 17L: 7D photoperiod. During the embryonic experiment, 30-50 embryos per replicate were reared in 0.9 l plastic vials containing 0.5 l treatment water. Embryos were reared from fertilization (day = 0) to day 12, i.e. when the larvae had hatched and reached the free-swimming stage upon leaving the egg capsule [ca. Gosner stage 20; [43]]. Survival of embryos was recorded during each water change (see below).

For the larval experiment, a randomly selected subset of tadpoles from each family was selected from the neutral embryonic treatment as soon as they had reached Gosner stage 25 (complete gill absorption and initiation of independent feeding, Gosner 1960), which occurred 12 - 15 days after fertilization. We used larvae from the neutral treatment because of high mortality in the acid treatments, which constrained the availability of healthy larvae and might have biased results due to selective mortality. Carry over effects from embryonic to larval stages have been shown to be small [44], suggesting that hatchlings transferred from pH 7.5 (embryonic neutral treatment) to pH 4.3 (larval acid treatment) are little affected by the change in pH. The tadpoles were randomly assigned to the two pH treatments and reared singly in 0.9 l opaque plastic containers containing 0.7 l treatment water. Tadpoles were fed *ad libitum *with finely chopped and parboiled spinach every second day. The amount of food was increased with increasing age of the tadpoles. When the tadpoles approached metamorphosis (emergence of at least one front leg; Gosner stage 42) the vials were checked daily.

### Response variables

Embryonic survival was recorded by visual inspection at each water change, but eggs were left untouched and only final survival (day 12) was used in the statistical analyses. Any unfertilized eggs were determined in conjunction to first water change (day 3) and were excluded from the analyses of survival. Embryos were considered dead if they did not hatch (i.e. escape the egg capsule) or were abnormal at hatching (e.g., bent spine, truncated body and oedema), as survival of abnormal hatchlings is very low [45]. Embryonic survival was therefore estimated as healthy hatched larvae at day 12/total number of fertilized eggs in each experimental unit.

In the larval experiment, survival was monitored at each water change, but only final survival was used in the statistical analyses. At metamorphosis, mass, larval period and average growth rate was estimated for each individual. Individuals were dry blotted and their wet mass measured with an electronic balance (to the nearest 0.1 mg). Larval period was estimated as the number of days from Gosner stage 25 (day 0 of larval experiment) to metamorphosis. Average daily growth rate (mg/day) was defined as the ratio of mass at metamorphosis/larval period.

Because maternal investment can differ among acid and neutral *R. arvalis *populations [46], and because egg size/initial size mediated maternal effects can affect larval performance of *R. arvalis *in a pH dependent way [21], average egg size of each parental female, as well as initial size (size at Gosner stage 25) of each experimental tadpole, was measured from digital images. For egg size, 20-40 eggs/female, and for initial size the individual stage 25 tadpole, were placed in a small petri dish, illuminated from below and photographed with a digital camera (Olympus Camedia C-5060 WideZoom with 5.1 megapixels). For photographing, eggs were covered with water and tadpoles placed in a thin layer of water to avoid dehydration whilst preventing movement. Egg size was measured as area (mm^2^) and initial size of tadpoles as total length from tip of the nose to tip of the tail (to the nearest 0.01 mm) using the public domain program ImageJ, version 1.39 u http://rsbweb.nih.gov/ij/.

### Statistical analyses

#### Phenotypic divergence

Both the embryonic and the larval experiment were performed as a nested randomized block design, where families (nested under pond pH) were used as random factors, and pond pH (eight levels), pH treatment (pH 4.0 and/or 4.3 and 7.5) and block (three levels) as fixed factors. For pond pH, average pond pH during the embryonic period was used in the embryonic analyses and average pond pH during the larval period in the larval analyses. As it is possible that the lowest pH (rather than the average pH) experienced by eggs or tadpoles in nature is a better predictor of acidity mediated selection, the same analyses were also run using minimum pond pH (instead of average pH). However, as mean and minimum pond pH are highly correlated (Persons *r *= 0.96, *N *= 8, *P *< 0.001) and the results in both analyses were very similar (data not shown), only analyses using mean pond pH are shown here.

All statistical analyses were conducted in SAS 9.2 (SAS Insitute, Inc.). Embryonic and larval survival were analyzed with generalized linear mixed models with binomial errors and logit link function in the GLIMMIX procedure [47]. Metamorphic mass, larval period and growth rate were log-transformed to homogenize variances and analyzed with mixed model analyses of (co) variance using the MIXED procedure [47].

Egg size and initial size were log-transformed to homogenize variances and analyzed using mixed models with pond pH as a fixed factor, and family as a random factor. The models for embryonic and larval traits were subsequently run with and without egg size (embryonic survival) or initial size (larval traits) as a covariate to control for potential contribution of egg size mediated maternal effects on offspring performance. As none of the interactions between fixed factors and the covariates were significant (*P *> 0.1), the final models only included the covariate main effects and not their interactions. Kenward-Roger degrees of freedom [47] were used in all analyses. To test whether breeding pond pH is linearly related to phenotypic trait values, linear orthogonal polynomials [48] were used to test for a linear relationship between pond pH and embryonic survival, larval life-history traits, egg size and initial size.

Taken together, a significant breeding pond pH main effect would indicate phenotypic divergence in trait means among populations, a significant pH treatment × pond pH interaction would indicate among population variation in pH tolerance (e.g., G × E interaction), and significant family and pH treatment × family interactions would indicate within population maternal/genetic variation. Any effects of egg or initial size would indicate that the responses might be modified by maternal effects. Linear effects of breeding pond pH on trait values would indicate a linear increase/decrease in the trait means along the acidity gradient, and that larger phenotypic differences correlate with larger environmental differences (i.e. presumably with relative strength of divergent selection). Interactions between linear effects of pond pH and pH treatment would indicate a linear increase/decrease in acid tolerance along the acidity gradient.

#### Putative agents of natural selection

In order to explore the relative contribution of pond pH (or directly correlated environmental variation) *versus *other habitat characteristics using phenotype-environment correlations, both environmental indices and individual variables were used. Calculation and results of the environmental indices are shown in Additional files 1, 2 and 3. All habitat variables (pond pH, water temperature, altitude, latitude, (log) pond size, canopy cover, predator density and tadpole density) were first tested for correlations among them (Additional file 4). To test for the relative importance of the different environmental variables, a set of models was produced where one environmental variable at a time was included as a covariate. These analyses were performed separately within each pH treatment and larval trait. Alternate models were conducted with either an environmental index (Additional file 2) or a relevant single environmental measure (e.g. pond pH or predator density). As all models were identical in other respects and contained only a single changing covariate, they were compared using *F *statistics [49]. In addition, AIC values were used to compare the different models [49]. The covariate with the highest *F *value (and the lowest AIC value) was considered to be most strongly correlated with a given larval trait. Population and family (nested within population) were both included as random effects to account for non-independence of experimental units and block as a fixed effect to account for a known temperature gradient within the laboratory.

## Results

### Phenotypic divergence

**Figure 2:**
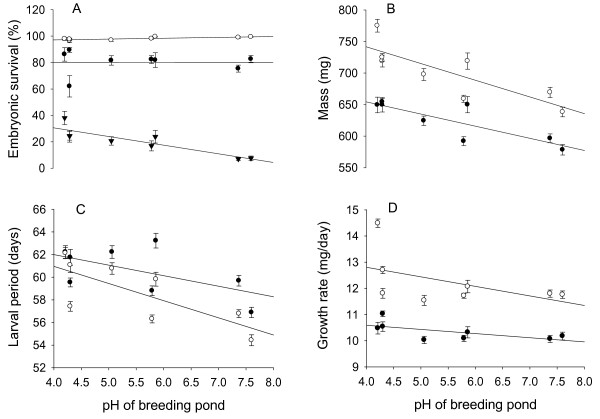
**Effects of the pH treatments on embryonic survival and larval traits for eight *R. arvalis *populations**. Raw data mean ± SE A) embryonic survival, B) metamorphic mass, C) larval period, and D) growth rate. The source pond pH is on the x-axis, and the different pH treatments are pH 7.5 (open circles), pH 4.3 (black circles) and pH 4.0 (black triangles).

*Embryonic survival *- Low pH severely reduced embryonic survival, as indicated by a significant pH treatment effect (Table 2, Figure 2A). However, populations differed strongly in their responses to pH, as indicated by a highly significant pond pH × pH treatment interaction (Table 2). Contrast results revealed that embryonic survival and pond pH were strongly *negatively *correlated at pH 4.0 (Figure 2A; *b *± S.E. = -1.86 ± 0.41, *P *< 0.001), not significantly correlated at pH 4.3 (Figure 2A; *b *= -0.28 ± 0.40, *P *= 0.484), and significantly *positively *correlated at pH 7.5 (Figure 2A; *b *= 1.07 ± 0.54, *P *= 0.048) - indicating that populations with highest embryonic survival in the acid treatment had slightly reduced survival in the neutral treatment. Embryonic acid tolerance (survival at pH 4.0) was significantly correlated with latitude (Additional file 4), most probably because of the strong spatial correlation between pond pH and latitude (Additional file 4). Using latitude instead of pond pH for the contrast analysis revealed that embryonic survival and latitude were strongly *negatively *correlated at pH 4.0 (*b *± S.E. = -1.81 ± 0.40, *P *< 0.001) but not significantly correlated at pH 4.3 (*b *= -0.13 ± 0.40, *P *= 0.744) or pH 7.5 (*b *= 0.64 ± 0.53, *P *= 0.232).

**Table 2:** Generalized linear mixed model of embryonic survival.

*Random effects*	*Variance ± SE*		*Z*	*P*
	
Family (Pond pH)	35.77 ± 14.78		2.42	**0.008**
Family (Pond pH) × pH treatment	80.88 ± 14.22		5.69	**< 0.001**
				
*Fixed effects*	*ndf*	*ddf*	*F*	*P*
	
Pond pH	7	60.8	1.2	0.319
pH treatment	2	127.4	430.6	**< 0.001**
Pond pH × pH treatment	14	120.2	2.7	**0.002**
Block	2	546.0	11.3	**< 0.001**
				
*Linear contrasts*	*ndf*	*ddf*	*F*	*P*
	
Pond pH	1	61.3	1.3	0.256
Pond pH × pH treatment (4.0 vs. 4.3)	1	85.6	10.6	**0.002**
Pond pH × pH treatment (4.0 vs. 7.5)	1	179.1	24.0	**< 0.001**
Pond pH × pH treatment (4.3 vs. 7.5)	1	174.2	5.2	**0.024**

Results are shown for eight *R. arvalis *populations occurring along a pH gradient. Significant effects are highlighted in **bold**

Significant family and family × pH treatment interactions indicated within population maternal effect/genetic variation in embryonic survival and pH tolerance (Table 2). Egg size differed significantly among populations (*F*_7, 63 _= 3.07, *P *= 0.008), but was not significantly correlated with pond pH (log *b *= -0.05 ± 0.04, *P *= 0.206). There was no significant egg size or pH treatment × egg size interaction in embryonic survival, and its inclusion as a covariate did not qualitatively change the results (not shown). This indicates that family effects in embryonic survival are independent of egg size.

*Larval traits *- Across all populations and treatments, larval survival was generally very high (ranging from 92.3% in Sätila to 98.8% in Lomsjö at pH 7.5, and from 84.2% in Sätila to 100% in Bergsjö and Rud at pH 4.3). There were no significant pond pH, pH treatment or their interaction effects on larval survival (all *P *> 0.40) and the data were therefore not further analyzed.

Metamorphic mass and growth rate decreased, and larval period increased at pH 4.3 compared to pH 7.5 (Figure 2B, C and 2D), as indicated by significant pH treatment main effects (Table 3). This indicates sub-lethal acid stress effects on tadpoles. Furthermore, populations have diverged in all three larval traits, as indicated by the significant pond pH main effects (Table 3). Significant linear contrasts further showed that metamorphic mass, larval period and growth rate increased with decreasing breeding pond pH (Table 3, Figure 2B, C and 2D). There was no significant pond pH × pH treatment effect on metamorphic mass or growth rate (Table 3 and 3). In contrast, larval period was more negatively affected by the acid treatment in neutral than in acid origin populations (ca. 2.5-3.5 days vs. 0-2 days delay, respectively; Figure 2C), giving rise to a significant pond pH × pH treatment interaction (Table 3). This suggests that acid origin populations have higher larval acid stress tolerance. The pH treatment dependent phenotypic divergence was also reflected in the relationship between pond pH and larval period: the correlation was stronger at pH 7.5 (log *b ± S.E*. = -0.09 ± 0.01, *P *< 0.001) than at pH 4.3 (log *b *± S.E. = -0.06 ± 0.01, *P *< 0.001; Figure 2C).

**Table 3:** Mixed model analysis of variance for larval traits.

	**a) Mass**				**b) Larval period**				**c) Growth rate**			
*Random effects*	*Var ± SE*		*Z*	*P*	*Var ± SE*		*Z*	*P*	*Var ± SE*		*Z*	*P*
			
Family (Pond pH)	2.58 ± 0.61		4.26	**< 0.001**	1.13 ± 0.28		4.09	**< 0.001**	2.95 ± 0.70		4.19	**< 0.001**
Family (Pond pH) × pH treatment	0		.		0.19 ± 0.11		1.72	**0.043**	0			
Residuals	9.99 ± 0.45		22.42	**< 0.001**	3.30 ± 0.15		21.79	**< 0.001**	12.42 ± 0.55		22.42	**< 0.001**
												
*Fixed effects*	*ndf*	*ddf*	*F*	*P*	*ndf*	*ddf*	*F*	*P*	*ndf*	*ddf*	*F*	*P*
			
Pond pH	7	55	7.7	**< 0.001**	7	55	8.4	**< 0.001**	7	55	2.8	**0.016**
pH treatment	1	1006	318.1	**< 0.001**	1	54	54.4	**< 0.001**	1	1006	427.4	**< 0.001**
Pond pH × pH treatment	7	1006	1.8	0.079	7	54	1.7	0.136	7	1006	0.7	0.661
Block	2	1006	2.4	0.094	2	955	0.2	0.835	2	1006	2.4	0.090
												
*Linear contrasts*	*ndf*	*ddf*	*F*	*P*	*ndf*	*ddf*	*F*	*P*	*ndf*	*ddf*	*F*	*P*
			
Pond pH	1	55	39.9	**< 0.001**	1	55	29.5	**< 0.001**	1	55	6.2	**0.016**
Pond pH × pH treatment	1	1006	1.7	0.194	1	54	6.6	**0.013**	1	1006	0.2	0.637

Results are shown for (log) a) metamorphic mass, b) larval period and c) growth rate in eight *R. arvalis *populations occurring along a pH gradient. Significant effects are highlighted in **bold**.

Significant family main effects indicated within population maternal effect/genetic variation in all three larval traits (Table 3; see discussion for the role of genetic and maternal effects in these populations). In addition, a significant pH treatment × family effect in larval period suggested within population maternal effect/genetic variation in pH tolerance for this trait. Populations (*F*_7,54 _= 2.23, *P *= 0.046) and families within populations (*Z *= 5.09, *P *< 0.001) differed significantly in initial size, but initial size was not significantly correlated with pond pH (log *b ± S.E *= -0.01 ± 0.02, *P *= 0.426). When initial size was included in the models of larval life-history trait variation as a covariate, it was significantly positively related to metamorphic mass (Additional file 5; log *b ± S.E*. = 0.27 ± 0.09, *F *= 8.19, *P *= 0.004) and growth rate (Additional file 5; log *b *= 0.42 ± 0.10, *F *= 17.80, P < 0.001), and significantly negatively related to larval period (Additional file 5; log *b *= -0.15 ± 0.06, *F *= 7.36, *P *= 0.007). These results indicate that initially large larvae grew and developed faster, but these effects were independent of pH treatment (non-significant initial size × pH treatment interaction). Moreover, the results remained qualitatively unchanged when initial size was included as a covariate (Additional file 5), indicating that family or population level responses were independent of initial size.

### Putative agents of natural selection

The analyses comparing effects of all single habitat variables (Table 4) or habitat indices (Additional file 3) found that the main correlates of phenotypic divergence were pH, latitude and predator density. In both treatments, metamorphic mass was most strongly, and negatively, related to pond pH (Table 4), and negatively, but more weakly, to latitude (Table 4). The pattern was different for larval period: larval period was most strongly, and negatively, related to latitude in both treatments (Table 4), and negatively, but more weakly, related to pond pH, and only in the high pH treatment (Table 4). These effects suggest that acidity may select for an increase in metamorphic size and climatic selection for a shorter larval period in the north. In contrast, larval growth was most strongly (positively) related to one of the habitat indices (i.e. habitat2, Additional file 3) and to predator density, and marginally (positively) to pond pH (Table 4). However, these relationships were apparent only at pH 4.3. These results suggest that predators, possibly together with canopy cover and/or pond pH, may be the most important selective factors behind divergence in growth rate.

**Table 4:** *F *tests and AIC values for larval traits from mixed model analyses of variance including single environmental variables as a covariate.

	a) Mass						b) Larval period						c) Growth rate				
	*F*	*AIC*	*P*	*100×* *Log(b)*	*100×* *SE*		*F*	*AIC*	*P*	*100×* *Log(b)*	*100×* *SE*		*F*	*AIC*	*P*	*100×* *Log(b)*	*100×* *SE*
*pH 4.3*																	
Pond pH	18.5	-799	**0.006**	-3.45	0.81		3.4	-1407	0.114	-1.66	0.90		4.9	-670	0.066	-1.88	0.85
Latitude	10.7	-795	**0.014**	-0.90	0.27		8.0	-1407	**0.028**	-0.59	0.21		1.3	-665	0.299	-0.32	0.29
Predator density	2.5	-791	0.175	0.90	0.57		0	-1403	0.904	0.06	0.48		10.5	-672	**0.002**	0.89	0.28
Canopy cover	0.2	-786	0.685	0.05	0.12		2.5	-1402	0.171	0.11	0.07		0.6	-662	0.479	-0.06	0.08
Altitude	0.8	-783	0.407	-0.02	0.02		6.0	-1400	0.053	-0.03	0.01		0.4	-659	0.571	0.01	0.01
Volume	0.8	-763	0.410	0	0		0.4	-1376	0.555	0	0		0.4	-638	0.550	0	0
Larval density	3.8	-793	0.100	-1.16	0.59		2.3	-1405	0.183	-0.68	0.45		1.1	-666	0.343	0.50	0.48
Temperature	0	-792	0.994	-0.02	2.66		0.6	-1406	0.469	1.35	1.74		0.7	-669	0.422	-1.49	1.75
																	
*pH 7.5*																	
Pond pH	18.4	-892	**0.007**	-3.96	0.92		7.4	-1417	**0.034**	-2.53	0.93		2.9	-793	0.142	-1.50	0.87
Latitude	9.8	-887	**0.022**	-1.02	0.32		14.9	-1417	**0.008**	-0.80	0.21		0.5	-789	0.499	-0.21	0.29
Predator density	0.5	-882	0.496	0.56	0.78		0.1	-1411	0.820	0.14	0.60		1.6	-790	0.272	0.49	0.39
Canopy cover	0.2	-878	0.646	0.07	0.14		1.7	-1409	0.241	0.12	0.09		0.5	-756	0.496	-0.06	0.08
Altitude	0.9	-875	0.390	-0.02	0.02		5.6	-1408	0.059	-0.03	0.01		0.6	-782	0.486	0.01	0.01
Volume	0.6	-855	0.457	0	0		0.5	-1384	0.503	0	0		0.2	-762	0.657	0	0
Larval density	3.5	-885	0.112	-1.33	0.71		3.2	-1413	0.127	-0.95	0.53		0.6	-790	0.454	-0.38	0.48
Temperature	0.1	-884	0.794	0.85	3.09		1.6	-1415	0.257	2.55	2.02		1.3	-793	0.299	-1.81	1.60

Results are shown for a) metamorphic mass, b) larval period and c) growth rate. Significant effects (*P *< 0.05) are highlighted in **bold**. The variable with the highest *F *value (and the lowest AIC value) is considered most important.

## Discussion

We found clear phenotypic divergence among *R. arvalis *populations along the acidification gradient: acid origin populations have higher embryonic acid tolerance (higher survival at acid pH), faster larval growth rates, longer larval periods, metamorphose at a larger size, and tend to have higher acid tolerance in terms of larval period than neutral origin populations. Our findings are in accordance with our previous studies on a smaller set of populations [13,21,25,50]. Phenotype-environment correlations further indicated that the extent of trait divergence correlates with the magnitude of environmental differences - and therefore presumably with strength of divergent selection [e.g., [30]]. However, a range of selective factors may be drivers of the observed phenotypic divergence. We next discuss evidence for adaptive divergence and the putative agents of selection along this acidification gradient, as well as the general implications of our findings for studies along environmental stress gradients.

### Evidence for adaptive divergence

Adaptive divergence along environmental gradients has been mainly studied in the context of adaptation to metal pollution [e.g., [10,51]] and climate [e.g., [52,53]], whereas studies along other stress gradients are rare. Our study adds to these studies, and provides further evidence for adaptation to acidification (zooplankton [e.g., [19]], fish [e.g., [20]] and amphibians [reviewed in [17]].

Local adaptation is expected to arise due to fitness trade-offs among contrasting environments [54]. In this vein, the increased embryonic survival of acid origin populations under acid conditions is clearly adaptive. In contrast, we found only marginal evidence for a trade-off between this increased acid tolerance and performance at neutral pH (populations with highest embryonic survival in the acid treatment had slightly reduced survival in the neutral treatment). Field transplant experiments suggest that the patterns for embryonic survival observed here under simple laboratory conditions also hold in the wild [50,55]. The reasons for this "asymmetric" adaptation are not clear, but could arise if fitness trade-offs become apparent only at extreme conditions (neutral conditions are generally benign) or in other contexts or traits [e.g., [3,56]]. Although the fitness consequences of the observed divergence in larval traits are less clear, large metamorphic size and short larval period are generally assumed to have a positive effect on fitness in amphibians [e.g., [22]]. Larval traits show evidence for trade-offs along the acidification gradient (i.e. acid origin populations were large but developed slower, whereas neutral origin populations were smaller but developed faster). Selection seems to favor different local optima along this acidification gradient, which suggests the potential for local adaptation in this system. Although more detailed studies and direct fitness estimates are needed to confirm local adaptation, the patterns observed here for embryonic and larval traits are strongly indicative of adaptive divergence and emphasize the importance of early life-history stages for adaptation [e.g., [11,13,57]].

In order for trait divergence to be an adaptation to divergent selection, it is necessary to confirm a heritable basis to the phenotypic traits [58]. We here used full-sib crosses on wild caught adults and reared embryos and larvae under laboratory common garden conditions, which does not allow to infer the genetic basis. However, several lines of evidence suggest that the observed patterns indeed reflect adaptive divergence. First, quantitative genetic crosses indicate that variation in embryonic acid tolerance is driven by maternal effects [25,26,50,59], which are clearly adaptive (embryos of acid origin mothers have higher acid tolerance). Whether these maternal effects have a genetic rather than environmental basis is work in progress. Second, divergence in larval traits is primarily due to direct genetic effects [59]. Reciprocal crosses between three population pairs [59], and within-population breeding experiments with half-sib (NCII) designs in a subset of four of the present populations (K. Räsänen and A. Laurila, unpubl. data), find evidence for direct genetic effects, negligible maternal effects and moderate to high heritability (*h^2 ^*up to 0.6) in larval traits. Furthermore, when we statistically controlled for initial larval size - indicative of maternal effects - the among population differences in trait divergence were maintained (Additional file 5). Third, correlations between the extent of phenotypic and environmental differences suggest that variation in trait divergence might reflect variation in the strength of divergent selection [e.g., [30]]. *Q*_ST_-*F*_ST _comparisons along this gradient [60] further indicate that the patterns of phenotypic divergence observed here are most likely a result of divergent natural selection rather than neutral processes, such as drift and gene flow [61]. We next turn to the discussion of the putative selective pressures along this acidification gradient.

### Agents of selection along the acidification gradient

Our phenotype-environment correlations, using several different abiotic and biotic environmental variables, found that trait divergence most strongly correlated with pond pH, latitude and predator density. Although we found a correlation between latitude and embryonic acid tolerance, we have no reason to expect increased selection or environmental influence for higher embryonic acid tolerance at lower latitudes. Moreover, divergence in embryonic acid tolerance is also seen between populations at similar latitudes [13]. Instead, we argue that this correlation most likely arose due to spatial autocorrelation between acidity and latitude, and that divergence in embryonic acid tolerance is most obviously driven by pond pH. This is also supported by the parallel patterns between laboratory and transplant experiments [50,55].

Complex life-history traits - such as larval growth and development - are likely under simultaneous selection from different sources [62]. In this vein, it is not surprising that our phenotype-environment correlations found that divergence in metamorphic mass is most closely related to pond pH, whereas divergence in larval period is most closely related to latitude, and larval growth to predator density, respectively.

Stressful (here acid) environments may also cause various forms of selection on stress tolerance and/or trait means [e.g., [3,56]], such as organismal growth and development rates [e.g., [6,63]]. Several hypotheses relating to larval life-history trait divergence on the acid-neutral axis were discussed in detail in [21] and we do not repeat them all here. For metamorphic mass, we suggest that large size may be more strongly selected for in acid environments to counteract the negative effects of acidic pH. Large size might be selected for due to increased fitness of large juveniles or adults during the terrestrial phase (e.g. either increased overwintering survival or reproductive success [e.g., [21,22]]).

For larval period, we found that development rate increased at higher latitudes, which is in accordance with countergradient selection [63]. However, in contrast to the commonly observed pattern in other Scandinavian *Rana *populations (e.g. 46, 47), we found no significant relationship between latitude and growth. We propose that in our study system, seasonal time constraints in the north favor faster development, whereas selection by predators (possibly in combination with canopy cover and/or pond pH) may drive divergence in growth rates. However, the relative contribution of climatic (latitude) and pH mediated selection on larval development is difficult to disentangle here as acidity and latitude were closely spatially correlated. Interestingly, populations with higher predator densities had higher growth rates, particularly in the low pH treatment. This could indicate selection for higher growth rates through gape limited predators [e.g., [64]], such as libellullid dragonflies, which are one of the most common predators of *R. arvalis *tadpoles in acidic ponds [[65], this study]. To shed light on this hypothesis, direct tests of predator mediated effects on larval performance are under way in our laboratory [59].

A few additional points need to be considered. First, our study provides correlative evidence for different selective agents acting on different fitness traits, and that different larval traits respond in part independently. As larval life-history traits can be phenotypically and genetically highly correlated [e.g., [66]], it would be interesting to study to what extent the different traits indeed respond independently to the different selective agents along environmental stress gradients [67]. Second, although we find strong correlations between some of our environmental variables and the extent of phenotypic divergence among local populations, there clearly is potential for several other factors to influence the extent of phenotypic divergence. These include variation in the extent of gene flow [e.g., [30,68]], the amount and type of genetic variation [e.g., [69]], as well genetic [e.g., [70]] and ecological trade-offs [e.g., [71]] among interacting selective forces. Our study was not designed to directly test for effects of individual habitat variables or other constraining factors to adaptation to acidity. Additional studies are therefore needed to better understand the relative importance of different selective or constraining factors to adaptive divergence in this system.

## Conclusions

We found evidence for phenotypic divergence among *R. arvalis *populations inhabiting an acidification gradient. As natural and anthropogenic environmental changes, such as acidification, result in a broad range of abiotic and biotic changes and interact with other geographically varying environmental factors (e.g. climate), it is important to investigate a range of putative selective factors. In line with this, acidity *per se *is the most likely direct selective factor behind divergence in embryonic acid tolerance, whereas divergence in larval life-history traits may be modified by interactions between pond pH, predator densities and climatic conditions - or factors closely correlated with them. This suggests that acidification interacts with local geographic variation in other selective factors in driving adaptive divergence of *R. arvalis*. As organisms need to optimize fitness in complex environments when faced with simultaneous selection by several abiotic and biotic factors [e.g., [72]], our findings emphasize the need to consider multiple interacting selective forces to understand the determinants of phenotypic divergence in natural populations and their responses to environmental change.

## Authors' contributions

SH, AL and KR designed the experiments. SH and KR collected the data and SH analyzed the data. SH wrote the manuscript and AL and KR corrected and revised earlier drafts. All authors read and approved the final draft of the manuscript.

## Supplementary Material

Additional file 1 **Calculation of habitat indices**. Click here for file

Additional file 2 **Details on the two principal component analyses and the resulting factor loadings**. Eigenvalues > 1 and factor loadings > |0.6| are highlighted in **bold**. Click here for file

Additional file 3 ***F *tests and AIC values for larval traits from mixed model analyses of variance including habitat indices as a covariate**. Results are shown for (log) a) metamorphic mass, b) larval period and c) growth rate. Significant effects (*P *< 0.05) are highlighted in **bold**. The variable with the highest *F *value (and the lowest AIC value) is considered most important. See Additional file 1 and 2 for description of habitat indices. Click here for file

Additional file 4 **Correlation matrix for habitat variables, embryonic survival and larval traits**. Significant Pearson *r *values (*P *< 0.05) are highlighted in **bold**. The correlations are based on population means (habitat variables: N = 9, larval traits: N = 8). Click here for file

Additional file 5 **Mixed model analysis of variance for larval traits including (log) initial size as a covariate**. Results are shown for (log) a) metamorphic mass, b) larval period and c) growth rate in eight *R. arvalis *populations occurring along a pH gradient. Significant effects are highlighted in **bold**. Click here for file
