# Supplementary material for: Adaptive divergence of the moor frog (Rana arvalis) along an acidification gradient
Source: BMC Evol Biol. 2011 Dec 19;11:366. doi: 10.1186/1471-2148-11-366 (PMC3305689; doi:10.1186/1471-2148-11-366)
Supplement: Additional file 1 — Calculation of habitat indices. [file 1471-2148-11-366-S1.DOC]

**Additional file 1 - Calculation of habitat indices.**

Environmental indices were calculated based on measured habitat characteristics using principal component analyses (PCA). Highly correlated variables were not included in the same analysis. Latitude was highly positively correlated with pond pH (Additional file 4) as more northern sites are situated on limestone bedrock and are therefore more neutral (Fig. 1A). Altitude was negatively correlated with temperature and positively correlated with tadpole density (Additional file 4). Because of these strong correlations, tadpole density and temperature were excluded from the PCA. The PCA included: predator density, (log) pond size, canopy cover, latitude and altitude. This gave three composite variables of habitat: habitat1-3. Habitat1 explained 40.4% of the variation and was mainly related to altitude, canopy cover, pond size and latitude (Additional file 2). Habitat2 explained 25.0% of variation and was mainly related to predator density and less to canopy cover. Habitat3 explained 20.3% of variation and was mainly related to pond size and latitude (Additional file 2).
