# Supplementary material for: Adaptive divergence of the moor frog (Rana arvalis) along an acidification gradient
Source: BMC Evol Biol. 2011 Dec 19;11:366. doi: 10.1186/1471-2148-11-366 (PMC3305689; doi:10.1186/1471-2148-11-366)
Supplement: Additional file 2 — Details on the two principal component analyses and the resulting factor loadings. Eigenvalues > 1 and factor loadings > |0.6| are highlighted in bold. [file 1471-2148-11-366-S2.DOC]

**Additional file 2 - Details on the two principal component analyses and the resulting factor loadings.**

| **PCA** | Eigenvalue | Proportion of variance explained | Cumulative variance explained |  | Factor loadings |  | |  | |  | |
| --- | --- | --- | --- | --- | --- | --- | --- | --- | --- | --- | --- |
|  | |  |  |  |  | Habitat1 | Habitat2 | | Habitat3 | |  |
| **1** (Habitat1) | **2.022** | 0.404 | 0.404 |  | Canopy cover | **-0.667** | **-0.640** | | 0.024 | |  |
| **2** (Habitat2) | **1.250** | 0.250 | 0.654 |  | Predators | -0.416 | **0.879** | | 0.141 | |  |
| **3** (Habitat3) | **1.014** | 0.203 | 0.857 |  | Pond size | **0.657** | -0.018 | | **-0.735** | |  |
| 4 | 0.613 | 0.123 | 0.980 |  | Latitude | **0.662** | -0.221 | | **0.651** | |  |
| 5 | 0.101 | 0.101 | 1.000 |  | Altitude | **0.731** | 0.132 | | 0.174 | |  |

Eigenvalues > 1 and factor loadings > ׀0.6׀ are highlighted in **bold**.
