# Supplementary material for: Adaptive divergence of the moor frog (Rana arvalis) along an acidification gradient
Source: BMC Evol Biol. 2011 Dec 19;11:366. doi: 10.1186/1471-2148-11-366 (PMC3305689; doi:10.1186/1471-2148-11-366)
Supplement: Additional file 3 — F tests and AIC values for larval traits from mixed model analyses of variance including habitat indices as a covariate. Results are shown for (log) a) metamorphic mass, b) larval period and c) growth rate. Significant effects (P < 0.05) are highlighted in bold. The variable with the highest F value (and the lowest AIC value) is considered most important. See Additional file 1 and 2 for description of habitat indices. [file 1471-2148-11-366-S3.DOC]

**Additional file 3 -  *F* tests and AIC values for larval traits from mixed model analyses of variance including habitat indices as a covariate.**

|  | a) Mass | | | | |  | b) Larval period | | | | |  | c) Growth rate | | | | |
| --- | --- | --- | --- | --- | --- | --- | --- | --- | --- | --- | --- | --- | --- | --- | --- | --- | --- |
|  | *F* | *AIC* | *P* | *Log(b)* | *SE* |  | *F* | *AIC* | *P* | *Log(b)* | *SE* |  | *F* | *AIC* | *P* | *Log(b)* | *SE* |
| *pH 4.3* |  |  |  |  |  |  |  |  |  |  |  |  |  |  |  |  |  |
| Habitat1 | 2.0 | -793 | 0.204 | -0.03 | 0.07 |  | 3.5 | -1408 | 0.113 | -0.02 | 0.01 |  | 0.1 | -667 | 0.774 | 0 | 0.01 |
| Habitat2 | 1.5 | -793 | 0.276 | 0.02 | 0.02 |  | 0.2 | -1406 | 0.715 | -0.01 | 0.02 |  | 12.7 | -677 | **0.007** | 0.03 | 0.01 |
| Habitat3 | 2.9 | -794 | 0.137 | -0.03 | 0.02 |  | 5.8 | -1409 | 0.052 | -0.02 | 0.01 |  | 0.1 | -667 | 0.775 | 0 | 0.01 |
|  |  |  |  |  |  |  |  |  |  |  |  |  |  |  |  |  |  |
| *pH 7.5* |  |  |  |  |  |  |  |  |  |  |  |  |  |  |  |  |  |
| Habitat1 | 1.5 | -885 | 0.267 | -0.03 | 0.02 |  | 4.5 | -1416 | 0.082 | -0.03 | 0.01 |  | 0 | -791 | 0.906 | 0 | 0.01 |
| Habitat2 | 0.3 | -884 | 0.586 | 0.02 | 0.03 |  | 0 | -1413 | 0.869 | 0 | 0.02 |  | 3.5 | -794 | 0.139 | 0.02 | 0.01 |
| Habitat3 | 3.5 | -887 | 0.110 | -0.04 | 0.02 |  | 5.0 | -1416 | 0.067 | -0.03 | 0.01 |  | 0.2 | 791 | 0.666 | -0.01 | 0.01 |

Results are shown for (log) a) metamorphic mass, b) larval period and c) growth rate. Significant effects (*P* < 0.05) are highlighted in **bold**. The variable with the highest *F* value (and the lowest AIC value) is considered most important. See Additional file 1 and 2 for description of habitat indices.
