# Supplementary material for: Adaptive divergence of the moor frog (Rana arvalis) along an acidification gradient
Source: BMC Evol Biol. 2011 Dec 19;11:366. doi: 10.1186/1471-2148-11-366 (PMC3305689; doi:10.1186/1471-2148-11-366)
Supplement: Additional file 4 — Correlation matrix for habitat variables, embryonic survival and larval traits. Significant Pearson r values (P < 0.05) are highlighted in bold. The correlations are based on population means (habitat variables: N = 9, larval traits: N = 8). [file 1471-2148-11-366-S4.DOC]

**Additional file 4 - Correlation matrix for habitat variables, embryonic survival and larval traits**.

|  |  | Canopy | Predator | Tadpole | Temp. | Pond | Altitude | pH | pH | Lat. | Embryonic survival | | | Mass | | Age | |
| --- | --- | --- | --- | --- | --- | --- | --- | --- | --- | --- | --- | --- | --- | --- | --- | --- | --- |
|  |  | cover |  | density |  | size |  | embryo | larvae |  | pH 4.0 | pH 4.3 | pH 7.5 | pH 4.3 | pH 7.5 | pH 4.3 | pH 7.5 |
| Canopy cover | | x |  |  |  |  |  |  |  |  |  |  |  |  |  |  |  |
| Predator | | -0.230 | x |  |  |  |  |  |  |  |  |  |  |  |  |  |  |
| Tadpole density | | -0.193 | -0.573 | x |  |  |  |  |  |  |  |  |  |  |  |  |  |
| Temperature | | 0.082 | 0.251 | -0.450 | x |  |  |  |  |  |  |  |  |  |  |  |  |
| Pond size | | -0.547 | -0.373 | **0.778** | -0.172 | x |  |  |  |  |  |  |  |  |  |  |  |
| Altitude | | -0.351 | -0.113 | **0.677** | **-0.680** | 0.497 | x |  |  |  |  |  |  |  |  |  |  |
| pH embryo | | 0.034 | -0.466 | 0.382 | -0.135 | 0.066 | -0.182 | x |  |  |  |  |  |  |  |  |  |
| pH larvae | | 0.074 | -0.539 | 0.484 | -0.244 | 0.118 | 0.253 | **0.988** | x |  |  |  |  |  |  |  |  |
| Latitude | | -0.350 | -0.377 | 0.496 | -0.258 | 0.239 | 0.394 | **0.881** | **0.872** | x |  |  |  |  |  |  |  |
| Embryonic survival | pH 4.0 | 0.152 | 0.158 | -0.419 | 0.092 | 0.247 | -0.187 | **-0.891** | **-0.880** | **0.825** | x |  |  |  |  |  |  |
|  | pH 4.3 | 0.031 | 0.097 | 0.125 | -0.082 | 0.062 | 0.494 | -0.024 | -0.029 | 0.047 | 0.202 | x |  |  |  |  |  |
|  | pH 7.5 | 0.175 | -0.557 | 0.287 | -0.536 | 0.029 | 0.331 | **0.733** | **0.760** | 0.622 | -0.432 | 0.323 | x |  |  |  |  |
| Mass | pH 4.3 | 0.208 | 0.459 | -0.643 | -0.041 | -0.352 | -0.350 | **-0.860** | **-0.854** | **-0.839** | 0.828 | -0.007 | -0.418 | x |  |  |  |
|  | pH 7.5 | 0.222 | 0.235 | -0.620 | 0.111 | -0.328 | -0.375 | **-0.836** | **-0.841** | **-0.775** | 0.943 | 0.123 | -0.378 | **0.904** | x |  |  |
| Age | pH 4.3 | 0.571 | 0.032 | -0.542 | 0.321 | -0.259 | **-0.722** | -0.607 | -0.603 | **-0.797** | 0.685 | -0.176 | -0.314 | **0.766** | **0.752** | x |  |
|  | pH 7.5 | 0.456 | 0.088 | -0.600 | 0.443 | -0.291 | -0.697 | **-0.753** | **-0.763** | **-0.864** | 0.815 | -0.218 | 0.539 | **0.766** | **0.843** | **0.918** | x |
| Growth | pH 4.3 | -0.291 | 0.694 | -0.418 | -0.398 | -0.281 | 0.239 | -0.660 | -0.657 | -0.419 | 0.526 | 0.208 | -0.297 | **0.709** | 0.578 | 0.092 | 0.175 |
|  | pH 7.5 | -0.192 | 0.372 | -0.330 | -0.390 | -0.218 | 0.269 | -0.520 | -0.517 | -0.270 | 0.609 | 0.550 | 0.025 | 0.630 | 0.674 | 0.133 | 0.174 |

Significant Pearson r values (P < 0.05) are highlighted in **bold**. The correlations are based on population means (habitat variables: N = 9, embryonic survival and larval traits: N = 8).
