# Supplementary material for: Adaptive divergence of the moor frog (Rana arvalis) along an acidification gradient
Source: BMC Evol Biol. 2011 Dec 19;11:366. doi: 10.1186/1471-2148-11-366 (PMC3305689; doi:10.1186/1471-2148-11-366)
Supplement: Additional file 5 — Mixed model analysis of variance for larval traits including (log) initial size as a covariate. Results are shown for (log) a) metamorphic mass, b) larval period and c) growth rate in eight R. arvalis populations occurring along a pH gradient. Significant effects are highlighted in bold. [file 1471-2148-11-366-S5.DOC]

**Additional file 5 - Mixed model analysis of variance for larval traits** **including (log) initial size as a covariate.**

|  | a) Mass | | | |  | b) Age | | | |  | c) Growth | | | |
| --- | --- | --- | --- | --- | --- | --- | --- | --- | --- | --- | --- | --- | --- | --- |
| *Random effects* | *Var ± SE* | | *Z* | *P* |  | *Var ± SE* | | *Z* | *P* |  | *Var ± SE* | | *Z* | *P* |
| Family (Pond pH) | 2.61 ± 0.62 | | 4.2 | **<0.001** |  | 0.97 ± 0.25 | | 3.9 | **<0.001** |  | 2.56 ± 0.64 | | 4.0 | **<0.001** |
| Family (Pond pH) × pH treatment | 0 | | . | **.** |  | 0.17 ± 0.11 | | 1.6 | 0.059 |  | 0 | | . | **.** |
| Residuals | 9.97 ± 0.45 | | 22.2 | **<0.001** |  | 3.30 ± 0.15 | | 21.6 | **<0.001** |  | 12.46 ± 0.56 | | 22.2 | **<0.001** |
|  |  | |  |  |  |  | |  |  |  |  | |  |  |
| *Fixed effects* | *ndf* | *ddf* | *F* | *P* |  | *ndf* | *ddf* | *F* | *P* |  | *ndf* | *ddf* | *F* | *P* |
| Pond pH | 7 | 55 | 6.8 | **<0.001** |  | 7 | 54 | 9.9 | **<0.001** |  | 7 | 54 | 2.8 | **0.014** |
| pH treatment | 1 | 986 | 313.1 | **<0.001** |  | 1 | 54 | 58.9 | **<0.001** |  | 1 | 987 | 423.9 | **<0.001** |
| Pond pH × pH treatment | 7 | 986 | 1.7 | 0.096 |  | 7 | 54 | 2.0 | 0.067 |  | 7 | 987 | 0.7 | 0.682 |
| Block | 2 | 987 | 2.5 | 0.084 |  | 2 | 936 | 0.1 | 0.917 |  | 2 | 987 | 2.4 | 0.092 |
| Initial size | 1 | 413 | 8.2 | **0.004** |  | 1 | 477 | 7.4 | **0.007** |  | 1 | 340 | 17.8 | **<0.001** |
|  |  | |  |  |  |  | |  |  |  |  | |  |  |
| *Linear contrasts* | *ndf* | *ddf* | *F* | *P* |  | *ndf* | *ddf* | *F* | *P* |  | *ndf* | *ddf* | *F* | *P* |
| Pond pH | 1 | 54 | 35.9 | **<0.001** |  | 1 | 54 | 31.9 | **<0.001** |  | 1 | 54 | 5.8 | **0.019** |
| Pond pH × pH treatment | 1 | 986 | 1.9 | 0.169 |  | 1 | 53 | 8.4 | **0.006** |  | 1 | 987 | 0.3 | 0.571 |

Results are shown for (log) a) metamorphic mass, b) larval period and c) growth rate in eight *R. arvalis* populations occurring along a pH gradient. Significant effects are highlighted in **bold.**
